# Supplementary material for: Activity-specific mobility of adults in a rural region of western Kenya
Source: PeerJ. 2020 Apr 29;8:e8798. doi: 10.7717/peerj.8798 (PMC7195828; doi:10.7717/peerj.8798)
Supplement: Supplemental Information 1 [file peerj-08-8798-s001.docx]

**Supplementary information for the manuscript “*Activity-specific mobility of adults in a rural region of western Kenya”***

Supplementary table 1: Relationships between travelling time and demographic characteristics of the surveyed population for different types of activity.

| Activity type | | | Explanatory variable | Estimate | p-value |
| --- | --- | --- | --- | --- | --- |
| Livestock activity | Travelling time | | Gender: male [Ref = female] | 1.43 [0.75, 2.86] | 0.287 |
|  |  |  | Occupation: non-farmer [Ref = farmer] | 1.50 [0.79, 2.81] | 0.215 |
|  |  |  | Household wealth | 2.25 [0.74, 6.84] | 0.162 |
|  |  |  | Age | 1.01 [0.99, 1.03] | 0.555 |
| Health facility | | Travelling time | Gender: male [Ref = female] | 0.80 [0.60, 1.09] | 0.153 |
|  |  |  | Occupation: non-farmer [Ref = farmer] | 0.90 [0.64, 1.29] | 0.559 |
|  |  |  | Household wealth | 0.94 [0.55, 1.60] | 0.824 |
|  |  |  | Age | 1.00 [0.99, 1.01] | 0.655 |
| Place of worship | | Travelling time | Gender: male [Ref = female] | 0.74 [0.51, 1.10] | 0.130 |
|  |  |  | Occupation: non-farmer [Ref = farmer] | 0.80 [0.52, 1.20] | 0.276 |
|  |  |  | Household wealth | 0.83 [0.47, 1.48] | 0.538 |
|  |  |  | Age | 1.00 [0.99, 1.01] | 0.940 |
| Market | | Travelling time | Gender: male [Ref = female] | 0.94 [0.45, 1.96] | 0.877 |
|  |  |  | Occupation: non-farmer [Ref = farmer] | 0.58 [0.28, 1.20] | 0.148 |
|  |  |  | Household wealth | 1.01 [0.40, 2.55] | 0.989 |
|  |  |  | Age | 0.99 [0.97, 1.02] | 0.574 |
| Household visits | | Travelling time | Gender: male [Ref = female] | 1.14 [0.83, 1.58] | 0.423 |
|  |  |  | Occupation: non-farmer [Ref = farmer] | 0.81 [0.59, 1.11] | 0.198 |
|  |  |  | Household wealth | 0.96 [0.64, 1.43] | 0.826 |
|  |  |  | Age | 1.01 [1.00, 1.02] | 0.074 |
| Water activity | | Travelling time | Gender: male [Ref = female] | 2.52 [0.92, 6.65] | 0.075 |
|  |  |  | Occupation: non-farmer [Ref = farmer] | 0.86 [0.34, 2.13] | 0.755 |
|  |  |  | Household wealth | 0.69 [0.22, 2.17] | 0.535 |
|  |  |  | Age | 1.00 [0.97, 1.03] | 0.976 |

**Household and individual surveys**

**Individual questions**

- Sublocation
- Household ID
- Collect the GPS coordinates of this household.
- Language of administration
- Name of respondent
- Age of respondent
- Gender of respondent
- Tribal origin
- Principal religion
- Marital status
- Level of education reached
- Years lived in current village
- Major occupation

**Individual movement questions**

Do you regularly visit any of these places?

- School
- Place of work (livestock-related)
- Place of work (other)
- Health facility
- Place of worship
- Market - animal
- Market - other
- Shop
- Another household
- Less regular, e.g. annual visits

For each place:

- Does the place have a name?
- How often do you typically go there?
- How do you usually travel there?
- How much does it cost?
- How long does it take you to get there (minutes)?
- How long do you typically spend there (hours)?
- Do any children go with you?
- How many?

**Household questions**

- How many members does this household have?
- Gender of household member
- Age of household member
- What is the highest school grade that the female head/spouse has completed?
- What is the main occupation of the male head/spouse?
- How many habitable rooms does this household occupy in its main dwelling?
- What is the floor of the main dwelling predominantly made of?
- What is your source of water for cooking?
- What is your source of water for drinking?
- What is the main source of lighting fuel for the household?
- Is there a latrine in the household?
- What type(s)?
- Is there evidence of scrounging by animals around the latrine?
- Transport: does your household own any of the following?
- Does your household own any irons (charcoal or electric?)
- How many mosquito nets does your household own?
- How many towels does your household own?
- How many frying pans does your household own?
- Do you grow crops?
- Why do you grow crops?
- How do you get to your crops?
- How much does it cost?
- Approximately how long does it take you to get to your crops in minutes?
- Where does the majority of your household access medical facilities?
- How do you normally travel to the medical facility?
- How long does it normally take to get there (minutes)?

**Household livestock questions**

- Does this homestead keep any animals?
- Where do you access veterinary services?
- Have you used the veterinary services in the last 12 months?
- Does this homestead keep cattle?
- Cattle questions
- How many males?
- How many females?
- Why do you keep cattle?
- Do you ever buy cattle or have you received cattle as a gift from outside the household?
- How long ago did you last buy/receive new cattle?
- Where do you usually buy/receive new cattle from?
- Do your cows/bulls engage in communal breeding?
- Are cattle herded with goats or sheep?
- How do you graze/feed your cattle?
- What is the water source for your cattle?
- Do you use medicine to prevent or treat disease in your cattle?
- Where do you get medicine for your cattle from?
- Have any cattle in the home been given any vaccinations?
- Do you know which vaccines have been given?
- Do you always pasteurize your milk before consumption?

**Cattle questions**

- Are you involved in feeding the cattle?
- Are you involved in milking the cattle?
- Are you involved in taking cattle to water?
- Are you involved in birthing the cattle?
- Have you ever experienced abortion in your herd?
- When was the last abortion?
- Do you ever handle cattle abortion material?
- What do you do with the aborted material?
- Are you involved in handling cattle manure?
- Do you ever buy cattle from a market?
- Do you ever take the cattle to a market?
- Are you involved in cattle skinning?
- Are you involved in cattle burial?
  - For each of the above:
  - How often do you do this activity?
  - Do you have to travel outside the household to do this activity?
  - What type of place do you go to for this activity?
  - If yes, how do you travel there?
  - How much does it cost?
  - How long does it take you to travel there (minutes)?
  - How long do you spend there (hours)?
  - How many children go with you?
  - What is the name of the place?

**Pig questions**

- Does this homestead keep pigs?
- How many piglets?
- How many adult males?
- How many adult females?
- Why do you keep pigs?
- Do you buy pigs from a market?
- What is the name of the market?
- How do you normally travel to the market?
- How much does it cost?
- How long does it normally take to get there (minutes)?
- How do you house the pigs?
- How do you feed the pigs?
- Are pigs fed waste?
- If pigs are fed waste, is it cooked prior to feeding it to the pig?
- Are pigs housed during any season?
- What is the flooring in the pig housing?
- Do you use medicine to prevent or treat disease in your pigs?
- Where do you purchase medicine for your pigs?
- Have the pigs on the homestead been vaccinated against anything?
- Does the participant know which vaccine was given to the pigs?
- Name/purpose of vaccine
- Are there any significant problems with your pigs?
- Are you involved in feeding the pigs?
- Are you involved in taking pigs to water?
- Are you involved in birthing the pigs?
- Are you involved in handling pig manure?
- Are you involved in taking pigs to market?
- Are you involved in pig slaughter?
- Are your pigs inspected at slaughter?
- Who inspects the meat for cysts?
- If cysts are found, what do you do with the meat?
- Are you involved in pig burial?
- For each of the activities above:
  - How often do you do this activity?
  - Do you have to travel outside the household to do this activity?
  - What type of place do you go to for this activity?
  - If yes, how do you travel there?
  - How much does it cost?
  - How long does it take you to travel there (minutes)?
  - How long do you spend there (hours)?
  - How many children go with you?
  - What is the name of the place?

**Sheep/goat questions**

- Does this homestead keep sheep/goats?
- How many sheep/goats?
- Do you buy sheep/goats from a market?
- What is the name of the market?
- How do you normally travel to the market?
- How much does it cost?
- How long does it normally take to get there (minutes)?
- How do you house the sheep/goats?
- How do you graze/feed your sheep/goats?
- Are you involved in feeding the sheep/goats?
- Are you involved in milking the sheep/goats?
- Are you involved in taking sheep/goats to water?
- Are you involved in birthing the sheep/goats?
- Are you involved in handling sheep/goat abortion material?
- Are you involved in handling sheep/goats manure?
- Are you involved in taking sheep/goats to market?
- Are you involved in sheep/goat slaughter?
- Are you involved in sheep/goat skinning?
- Are you involved in sheep/goat burial?
- For each of the activities above:
  - How often do you do this activity?
  - Do you have to travel outside the household to do this activity?
  - What type of place do you go to for this activity?
  - If yes, how do you travel there?
  - How much does it cost?
  - How long does it take you to travel there (minutes)?
  - How long do you spend there (hours)?
  - How many children go with you?
  - What is the name of the place?

**Final questions**

- Does this homestead keep chickens?
- Do you feed your chickens?
- Do livestock have access to the buildings you sleep in?
- Which livestock have access to the buildings you sleep in?
- In the last 12 months, have you seen wildlife around the home?
- What wildlife have you seen?
- Tracker given?
- Tracker ID
